# Phosphorus fractions in soil fertilised with organic waste

**DOI:** 10.1007/s10661-020-8190-9

**Published:** 2020-04-27

**Authors:** Jadwiga Wierzbowska, Stanisław Sienkiewicz, Marta Zalewska, Piotr Żarczyński, Sławomir Krzebietke

**Affiliations:** Department of Agricultural Chemistry and Environmental Protection, University of Warmia and Mazury in Olsztyn, Oczapowskiego 8, 10-744 Olsztyn, Poland

**Keywords:** Composts, Phosphorus in the soil, Organic fertilisation, Waste organic substances

## Abstract

The aim of this study has been to evaluate the effect of sewage sludge and composted sewage sludge and municipal waste on the content of various forms of P in soil. The experiment scheme: C, control; NPK; FYM; DGSS, dried and granulated sewage sludge; CSS, composed sewage sludge; CSSS, composted sewage sludge and straw; CMMW, composted mixed municipal waste; CMGW, composted municipal green waste. The content of bound P was determined in the fractions: F1, easily soluble; F2, exchangeable; F3, organic; F4, carbonate; F5, stable organic-mineral and mineral bonds; and F6, residual. The NPK fertilisation as well as the soil fertilisation with organic substances raised the P-total content and of P bound in the fractions: F3, F4, F5 and F6. The highest amount of phosphorus in the studied soil was in fraction F3 (phosphorus in organic compounds) and the lowest in fraction F1 (phosphorus in the ionic form as H_2_PO_4_^−^ and HPO_4_^2−^). Composted sludge and straw introduced into the soil increased the content of readily soluble P (F1), while the NPK effect was reversed. NPK fertilisation and enhancement of soil organic matter (except CSSS, CMGW) led to a reduction of the P content in F2 fraction. The content of available P determined by the Egner-Riehm method depended on the content of C-organic, P-total and CEC soil. Among the determined phosphorus fractions, the content of available P was most strongly correlated with the content of P bound in the carbonate fraction (F4) and residual fraction (F6) and, less strongly, with the organic phosphorus fraction.

## Introduction

Phosphorus is an essential element for the growth of living organisms, but when present in soil in excessive quantities, this element can contribute to the eutrophication of water bodies (Devau et al. 2011; Kahiluoto et al. 2015). Both in agronomy and in environmental sciences, rational soil management requires knowledge of the forms of phosphorus occurring in soil (Cordell et al. 2009), as this information is important for diagnosing the bioavailability and mobility of phosphorus in soil (González Medeiros et al. 2005). The content of phytoavailable forms of phosphorus can be determined with speciation analysis, which encompasses procedures that enable both qualitative and quantitative identification of phosphorus forms (Cabeza et al. 2019). The knowledge of the total content of phosphorus as well as its fractions in soil can also aid the identification of a eutrophication threat in the environment (Esteller et al. 2009; Sapek 2014), because each fraction of this element is different with regard to its potential mobilisation and circulation in nature (Pakuła and Kalembasa 2008).

Soil contains both organic (P-organic) and mineral (P-mineral) forms of phosphorus. Most of the total P amount is composed of weakly soluble phosphorus fractions, unavailable or poorly available to plants (Murphy and Sims 2012). The content and stability of P-organic are positively correlated with the organic matter content but correlate negatively with the content of Al and Fe. Organic phosphorus compounds become available to plants only after organic matter has been mineralised, leading to an increase in the soil content of mineral phosphorus forms, which become phytoavailable and return to the P circulation in soil (Noack et al. 2012; Wang et al. 2012.). In its mineral form, phosphorus most often binds to iron or aluminium, a process associated with the prevalence of acidic soils, favouring the retrogradation of phosphorus to these compounds (Potarzycki 2000).

It is estimated that phosphate resources will be depleted in 50–100 years, should the current level of their excavation be maintained. This means that the deficit of phosphorus could become the main crop production limiting factor. Sewage sludge and other organic waste are a source of numerous biogens, including phosphorus (Singh and Agrawal 2008; Czechowska-Kosacka 2016; Wierzbowska et al. 2016a; Foereid 2017; Sienkiewicz et al. 2018). The organic substances used to fertilise soil contain phosphorus in different speciation forms, whose type and quantities depend on the physicochemical conditions set up for wastewater and sewage processing (Bezak-Mazur and Mazur 2011; Czechowska-Kosacka 2016) or on the chemical composition of composted matter (Grigatti et al. 2015; Jakubus 2016). The type of a natural fertiliser plays an important role in both the short-term availability of phosphorus to plants and the long-term speciation of phosphorus compounds (Kar et al. 2017). According to Abolfazli et al. (2012), organic fertilisation changes the physicochemical properties of soil and raises the soil solution content of phosphorus. Organic matter contained in manure or composted municipal waste can enhance the accessibility of phosphorus to plants by limiting its retrogradation, and additionally, when undergoing mineralisation, it increases the supply of soluble, plant-available form of this element.

The aim of this study has been to evaluate the effect of sewage sludge and composted sewage sludge and municipal waste on the content of various forms of phosphorus in soil.

## Material and methods

A field trial was conducted in 2004–2015, in Bałcyny (53°35′49″N 19°51′20″E). The experiment was set up on grey-brown podzolic soil underlain by light glacial till (Iuss Working Group WBR 2015). The trials spanned 3 crop rotation cycles: potato, spring barley, winter oilseed rape and winter wheat. Before the experiment began, the soil had the following properties: C-organic content of 7.63 g/kg; N-total, 0.64 g/kg; and content of available forms of P, K and Mg 45.10, 132.30 and 48.30 mg/kg, respectively. The soil was slightly acidic in reaction (pH in 1 mol KCl/dm^3^ was 5.70), and its hydrolytic acidity equalled 27.70 mmol^(+)^/kg.

During the 12 years of the field trials, the soil was enriched with 30 t DM/ha of organic matter, i.e. 10 t DM/ha in each crop rotation. All organic materials were used in the form of fresh mass in an amount corresponding to 10 t DM/ha per rotation. All variants (except the control) were fertilised with N, P and K in doses corresponding to the crop’s requirements (processing potato, N-150, P-28, K-166; spring barley, N-90, P-26, K-100; winter oilseed rape, N-120, P-42, K-134; winter wheat, N-90; P-26; K-100 kg/ha). In the years when manure and other organic substances were used as fertilisers, depending on their N content, the dose of this element was supplemented up to the level of 150 kg N/ha with the use of ammonium nitrate. Soil samples for the 0–20 cm horizon were taken after the third rotation cycle finished, i.e. after winter wheat was harvested in 2015.

The experiment was composed of the following treatments: C, control (no fertilisation); NPK; FYM, mixed manure; DGSS, dried and granulated sewage sludge; CSS, composed sewage sludge; CSSS, composted municipal sewage sludge and straw; CMMW, the Dano compost made from mixed municipal waste; and CMGW, composted municipal green waste. The tested organic substances used for soil fertilisation are available to farmers free of charge.

The content of dry matter in organic materials was determined by the oven-drying method (PN-EN 15934:2013-02E), and the content of organic carbon was assayed after drying samples in a muffle oven at a temperature of 520 °C (PN-EN 15936:2013-02E). Having mineralised the organic substances in concentrated sulphuric acid, the content of N-total was determined with the Kjeldahl method (on a KjelFlex K-360 apparatus), and the phosphorus content was measured by *phosphorus vanadium molybdenum* yellow *spectrophotometry* (on a Shimadzu UV 1201 V).

The soil pH was determined potentiometrically in a solution of 1 mol KCl/dm^3^, and the hydrolytic acidity of the soil was determined with the Kappen method (Tyszkiewicz et al. 2019). The soil adsorption complex capacity was calculated as the sum of alkaline cations (determined after extraction with 1.0 mol CH_3_COONH_4_/dm^3^) and hydrolytic acidity (Ostrowska et al. 1991). The content of available phosphorus was determined with the Egner-Riehm method (PN-R-04023. 1996), while that of organic carbon was identified in a Vario Max Cube CN Elementar analyser.

Division of phosphorus compounds in soil into fractions followed the method developed by Hedley and modified by Tiesser and Moir (1993). The ratio of soil to extraction solutions was 1:10. The content of bound phosphorus was determined in the following fractions:

- F1 – Easily soluble fraction (phosphorus in the ionic form as H_2_PO_4_^−^ and HPO_4_^2−^) extracted with deionised water
- F2 – Exchangeable fraction (phosphorus bound exchangeably, adsorbed nonspecifically to the soil’s solid phase), extracted with 0.5 mol/dm^3^ NaHCO_3_
- F3 – Organic fraction (phosphorus in organic compounds), extracted with 0.1 mol/dm^3^ NaOH
- F4 – Carbonate fraction (phosphorus in carbonates), extracted with 1 mol/dm^3^ HCl
- F5 – Stable organic-mineral and mineral bonds (P-Al, P-Fe, P-Mn), extracted with concentrated HCl
- F6 – Residual fraction – the difference between the total P content in soil and the sum of determined fractions (F6 = Pt - (F1 + F2 + F3 + F4 + F5).

An amount approximately equal the total phosphorus content in soil was extracted in a mixture of perchloric acid and nitric acid (Ostrowska et al. 1991). The content of phosphorus in particular fractions was determined by colorimetry on a Shimadzu UV 1201 V apparatus, applying the molybdenum method (ammonium molybdate with the addition of tin (II) chloride as a reducer).

Results of all chemical analyses were processed statistically with the help of a software package Statistica 13.3®. The Tukey’s test at a significance level of α = 0.05 served to verify the significance of differences and also calculated coefficients of correlation *r* between selected properties of the soil and organic waste and the content of determined phosphorus fractions.

## Research results and discussion

The organic substances used for soil fertilisation varied not only in the dry matter or total nitrogen content but also in the content of phosphorus (P) and the C/P ratio and C/N ratio values (Table 1). Compared to manure (FYM), all other organic substances contained more P and had a narrower C/P ratio. The lowest C/N ratio value was identified in composted sewage sludge (CSS) – 7.3 – whereas dried and granulated sewage sludge (DGSS) was found to have a 1:20.3 C/N ratio. The biggest amounts of P were incorporated into the soil with CSS and with DGSS (199.3 and 122.7 kg, respectively, over each of the 4-year crop rotation cycle), while the lowest quantity of this element was added to soil with mixed municipal waste compost (CMMW) and with urban green compost (CMGW) (about 10% less than with FYM).

**Table 1:** Content and load of macroelements introduced to soil with organic materials

Elements	Manure (FYM)	Dried and granulated sewage sludge (DGSS)	Composted sewage sludge (CSS)	Composted municipal sewage sludge and straw (CSSS)	The “Dano” compost made from mixed municipal waste (CMMW)	Composted municipal green waste (CMGW)
Content (g/kg)						
Dry matter (DM)	222.4	851.4	403.1	586.8	746.4	788.8
Content (g/kg DM)						
C-org.	76.2	325.8	86.2	108.4	101.7	63.3
N-tot.	5.80	16.02	11.77	6.28	6.94	4.50
P-tot.	1.02	10.45	8.03	4.41	2.94	3.23
C/N	13.1	20.3	7.3	17.2	14.6	14.1
C/P	74,7	31,2	10,7	24,6	34,6	19,6
Load (kg/ha per rotation)						
C-org.	3426.3	3827.0	2139.0	1847.0	1362.0	802.0
N-tot.	260.8	188.2	292.0	107.5	93.0	57.0
P-tot.	45.0	122.7	199.3	75.2	39.4	41.0

**Fig. 1:**
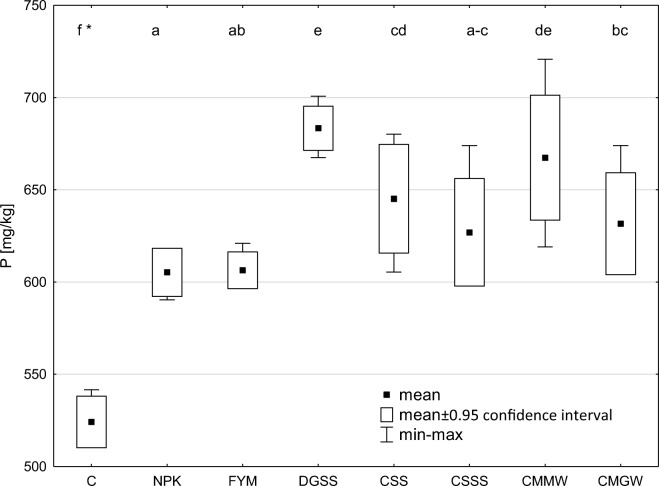
Content of total phosphorus in soil (*data marked with the same letters do not differ significantly at *p* < 0.05; C, control; NPK; FYM, manure; DGSS, dried and granulated sewage sludge; CSS, composed sewage sludge; CSSS, composted municipal sewage sludge and straw; CMMW, the Dano compost made from mixed municipal waste; and CMGW, composted municipal green waste)

The lowest P-total content (524.2 mg/kg) was determined in soil of the control soil (Fig. 1). The P-total content was similar in the soil fertilised with mineral fertilisers alone and in the manure-treated soil (605.2 in the former and 606.3 mg/kg in the latter case).The highest P-total in soil was detected in the treatments with DGSS and with CMMW (686.3 and 667.4 mg/kg, respectively).

**Fig. 2:**
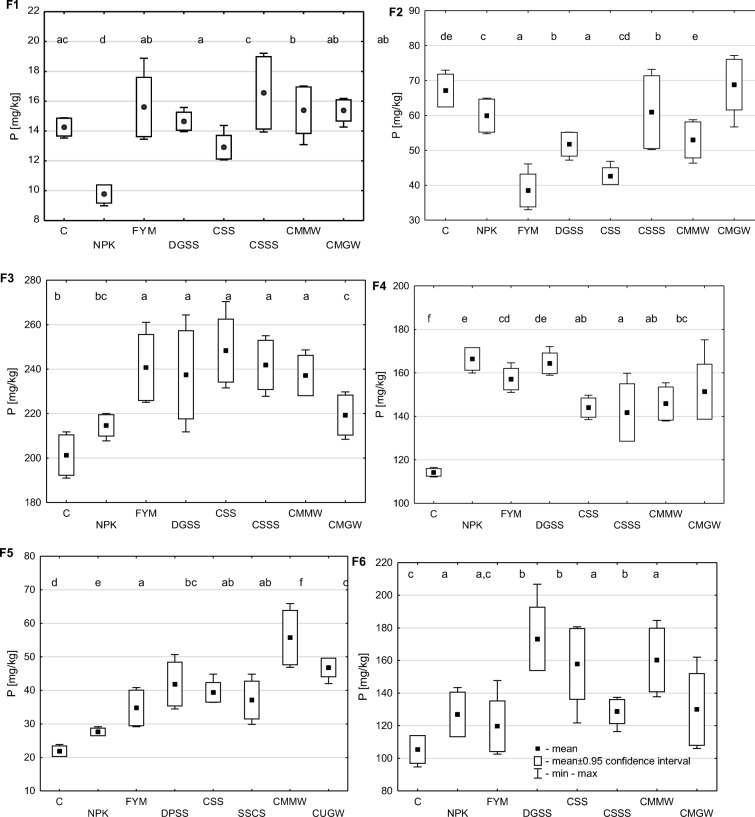
Content of phosphorus bound in the determined fractions (*data marked with the same letters do not differ significantly at *p* < 0.05; C, control; NPK; FYM, manure; DGSS, dried and granulated sewage sludge; CSS, composed sewage sludge; CSSS, composted municipal sewage sludge and straw; CMMW, the Dano compost made from mixed municipal waste; and CMGW, composted municipal green waste; F1, easily soluble fraction; F2, exchangeable fraction; F3, organic fraction; F4, carbonate fraction; F5, stable organic-mineral and mineral bonds; F6, residual fraction)

The data shown in Fig. 2 demonstrate that the content of bound P in the distinguished fractions decreased in the following order: F1 < F5 < F2 < F4 < F6 < F3. The content of P in the easily soluble fraction (F1) ranged from 9.78 to 16.56 mg P/kg. The least P in this fraction was found in the soil fertilised solely with mineral NPK (> 30% less than in the control soil). Compared to the exclusively NPK-fertilised variant, composts and sewage sludge raised the share of this phosphorus fraction by 32% (CSS) to 69% (CSSS). The lowest amount of P bound in the exchangeable fraction (F2) was determined in soil fertilised with FYM or with CSS (38.50 and 42.61 mg P/kg, respectively), while the highest quantities of this fraction’s P were observed in soil fertilised with CMGW and in soil from the control soil (68.81 and 67.13 mg P/kg, respectively). Compared to the control soil (201.3 mg P/kg) and soil fertilised only with mineral fertilisers (214.7 mg P/kg), the organic substances added to soil raised the content of P in the organic fraction (F3). Most P in this fraction was contained in the soil fertilised with CSS, CSSS and FYM (248.4, 241.9 and 240.7 mg P/kg, respectively). The least of P bound in the carbonate fraction (F4) was determined in the control soil (114.2 mg P/kg). Mineral and organic fertilisation significantly increased the content of this P fraction in soil. Most P of fraction F4 was noted in the soil fertilised with NPK alone or with DGSS (over 45% more than in the control soil), while the smallest increase in its content (about 24%) was determined in the soil fertilised with CSSS. The lowest content of P in stable organic-mineral and mineral bonds (F5) was found in the control soil (21.76 mg P/kg). Fertilisation, especially with organic substances, increased the soil content of this P fraction. Most P of fraction F5 was observed in the soil fertilised with CMMW (about 2.5-fold more than in the control soil). Compared to the control soil, mineral as well as organic-mineral fertilisation raised the content of P bound in the residual fraction (F6). The content of this form of P in the control soil was 105.38 mg P/kg. Most fraction F6 phosphorus was determined in the soil fertilised with DGSS (173.20 mg P/kg), CSS (157.85 mg P kg^−1^) or CMMW (160.25 mg P/kg). Among the tested organic substances, the weakest impact on the content of P accumulated in the residual fraction was produced by FYM (119.65 mg P/kg).

In a study by Majchrowska-Safaryan et al. (2017), spent mushroom substrate used as a soil fertiliser significantly increased the content of phosphorus in the organic fraction (F3). According to Redel et al. (2013), soils fertilised with large doses of organic fertilisers do not necessarily contain more P in the organic fraction than the ones not fertilised with organic substances. Braos et al. (2015) maintain that fertilisation with cattle manure only moderately raised the content of labile as well as permanent bonds of P-organic in soil. Soluble forms of inorganic phosphorus had a stronger effect than instable bonds of this element on the availability of phosphorus to plants. Kalbasi and Karthikeyan (2004) concluded that fertilisation of slightly acidic soils with natural and mineral fertilisers carried out for several years resulted in phosphorus being accumulated mainly in compounds with aluminium. On the other hand, Kalembasa and Kuziemska (2007) suggested that sewage sludge introduced to soil raised the soil content of P-total, and the highest share of extracted phosphorus consisted of P bound with Al, while the smallest percentage was contained in occluded phosphates.

Galvăo and Salcedo (2009) were of the opinion that long-term application of natural fertilisers led to the accumulation of P in soil, with inorganic forms prevailing over the organic ones. In turn, Kashem et al. (2004) suggest that application of cattle manure could decrease the soil content of soluble phosphorus, concurrently with an increase in the less stable forms of P. This dependence is attributed to the activity of soil microorganisms converting labile phosphorus compounds into organic compounds, which in turn is triggered by a higher amount of harvest residue left by manure fertilised crops, as well as the broad C/P ratio in this fertiliser. According to Tiecher et al. (2017), a rise in the P-organic content is restrained by the amount of C-organic in soil. Generally, an increase in P-total is accompanied by greater accumulation of labile fractions of this element, and their quantities depend on the N:P ratio and on the content of P in the fertiliser. It is not until the subsequent stage that the content of P bound in the acid-soluble fraction increases (Ca-P). Persistent application of manure can lead to the migration of P-organic into deeper soil horizons. The presence of easily soluble minerals in soil (CaHPO_4_ 2H_2_O) can increase the leaching of phosphorus with percolating water (Kar et al. 2017). In another study by Wierzbowska et al. (2016b), most P-total leached from soil treated with manure (373.5 μg/dm^3^), while the smallest amount was leached from NPK-fertilised soil (77.9 μg/dm^3^). The content of P-PO_4_^3−^ in leachate was from 67.5 μg/dm^3^ after soil had been fertilised with manure to 118.9 μg/dm^3^ when it had been enriched with dried and granulated manure. The share of P-PO_4_^3−^ in the total phosphorus content in leachate ranged from 25 to 87%. Mineral P is not very mobile in soil; therefore, it can be suspected that organic minerals contribute profoundly to the leaching of this component. Couto et al. (2017) also demonstrated that soil application of organic waste continued for many years led to the accumulation of labile P forms in soil (P extracted with ion-exchange resin and 0.5 mol/dm^3^ solution of NaHCO_3_) to a level much above the quantity needed for proper nourishment of plants or above the soil’s retention capacity concerning this element and therefore environmental pollution was a likely consequence. The authors maintain that setting doses of organic waste to be applied for soil fertilisation only in accordance with the crops’ nutritional requirements is ecologically incorrect. According to Kahiluoto et al. (2015), phytoavailable of P from properly treated waste may be even better than in NPK. The organic substance contained in the organic waste prevents phosphorus sorption in the soil and enables its easier uptake by plants. The more so, according to Djodjic and Bergström (2005), significant losses of P as a result of leaching apply not only to fertilised fields or fields with P contents in soil above the agronomic optimum. Similarly, Lourenzi et al. (2015) point out that as a result of the use of pig sewage, the danger of P being washed out of soil increases in the following order: available P>soluble P>particulate P. Pietrzak et al. (2013) reported that chicken manure application resulted in excess of P in the soil and it was concluded that phosphorus could be present in surface runoff in enhanced concentration. They also found out that the content of available phosphorus in the upper 5 cm soil layer was positively correlated with its concentration in surface runoff.

**Fig. 3:**
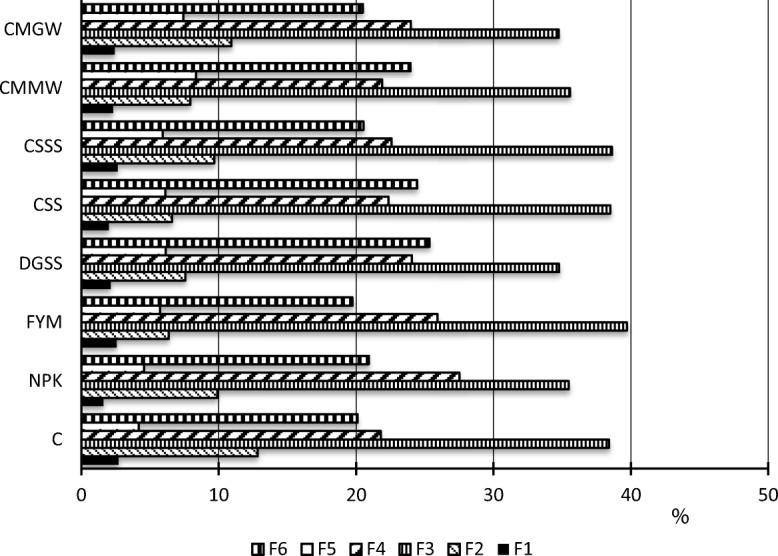
Share of P bound in the distinguished fractions (%) (NPK; FYM, manure; DGSS, dried and granulated sewage sludge; CSS, composed sewage sludge; CSSS, composted municipal sewage sludge and straw; CMMW, the Dano compost made from mixed municipal waste; and CMGW, composted municipal green waste; F1, easily soluble fraction; F2, exchangeable fraction; F3, organic fraction; F4, carbonate fraction; F5, stable organic-mineral and mineral bonds; F6, residual fraction)

The analysed soil samples were found to be highly diverse in terms of the content of P bound in the distinguished P fractions (Fig. 3), which was as follows: F3 (34.7–39.7%)>F4 (21.8–27.5%)>F6 (19.7–25.3%)>F2 (6.5–12.8%)>F5 (4.2–8.4%)>F1 (1.6–2.7%). In comparison to the control soil, the application of exclusive mineral fertilisation decreased the share of easily soluble P (F1) while increasing the share of carbonate fraction phosphorus (F4) by about 6%. The soil fertilised with FYM or with CSS was observed to have the exchangeable fraction P (F2) lower by nearly half. Relative to the soil treated with FYM, the soil fertilised with DGSS or CMGW resulted in an approximately 5% decline in the share of P bound in the organic fraction (F3). Fertilisation, and particularly the incorporation of municipal waste to soil, favoured the binding of P into stable organic and mineral bonds (F5). The smallest share of the residual fraction P (F6) was identified in the manure-treated soil, while the soil incorporation of sewage sludge, either dried and granulated or composted, increased the share of this P fraction in soil by about 25%.

Whalen et al. (2001) as well as He et al. (2006) maintain that the dynamics of transformations of mineral and organic phosphorus compounds in soil depends on the soil’s properties shaped under the influence of long-term fertilisation. In a study by Adhami et al. (2013), the soil’s pH had a nearly linear negative relationship with the content of phosphorus bound to aluminium and iron (NaOH-P) and a linear positive relationship with the content of phosphorus in the fraction of lithogenic apatites (HCl-P). Bednarek et al. (2017) state that phosphorus applied in the form of triple superphosphate is converted in soil mainly into fractions of iron and aluminium phosphates and, to a lesser degree, into the calcium phosphate fraction, while its contribution to the fraction of easily soluble phosphates is the smallest. According to Jakubus (2015), it is the type of soil that decides which phosphorus fraction becomes dominant. In gleic soils, the highest percentage is composed of permanently bound phosphorus (58% of P-total), whereas in organic soils the dominant fraction was that of organic phosphorus (49% of P-total). In these soils, the content of organic matter and total and available phosphorus have a strong influence on the content of water-soluble P. Jokubauskaitė et al. (2015) concluded that when the soil’s pH reached the neutral reaction owing to soil liming, its content of water-soluble and plant available P increased.

The data shown in Table 2 demonstrate that the analysed soil properties did not have a significant influence on the content of easily soluble P (F1) or P bound exchangeably (F2). On the other hand, the organic fraction P (F3) content was positively correlated with the soil adsorption complex capacity (*r* = 0.29*), and the widening of the C/P ratio affected negatively (*r* = − 0.33*) the content of this fraction’s P. The content of P in the carbonate fraction (F4) was most strongly correlated with the P-total content (*r* = 0.60*) but showed a weaker correlation with the adsorption complex capacity or the C-organic content (*r* = 0.54*; *r* = 0.48*, respectively). The content of P in stable organic-mineral bonds (F5) was positively correlated with the P-total content and the soil’s pH (*r* = 0.60* and *r =* 0.42*, respectively) while being negatively correlated with hydrolytic acidity (Hh) and the C/P ratio (*r* = − 0.29* and *r* = − 0.41*, respectively). Finally, the content of P bound in the residual fraction (F6) depended above all on the P-total content in soil (*r* = 0.81*) and slightly less strongly on the content of C-organic and the C/P ratio (*r* = 0.47* and *r* = 0.38*, respectively).

**Table 2:** Correlation coefficients *r* between selected soil properties and the content of the determined phosphorus fractions

Selected soil properties	Phosphorus fractions						P-available
	F1	F2	F3	F4	F5	F6	
pH	0.17	− 0.13	0.08	0.27	0.42*	0.09	0.27
Hh	− 0.14	0.12	− 0.01	− 0.17	− 0.29*	0.16	0.06
CEC	− 0.20	− 0.24	0.29*	0.54*	0.24	0.03	0.40*
C-org.	0.16	− 0.20	0.07	0.48*	0.23	0.47*	0.66*
P-tot.	0.01	− 0.16	0.23	0.60*	0.60*	0.81*	0.80*
C/P	0.14	− 0.02	− 0.33*	− 0.23	− 0.41*	0.38*	− 0.27
P-available	− 0.08	− 0.18	0.32*	0.65*	0.28	0.60*	–

*CEC* cation exchange capacity, *Hh* hydrolytic acidity, *P-available* P determined with the Egner-Riehm method; *significant at *p* < 0.05, *n* = 48; *F1* easily soluble fraction, *F2* exchangeable fraction, *F3* organic fraction, *F4* carbonate fraction, *F5* stable organic-mineral and mineral bonds, *F6* residual fraction

The content of available P in soil determined with the Egner-Riehm method was significantly positively correlated with the content of organic carbon, content of P-total and capacity of the soil adsorption complex (*r* = 0.66*, *r* = 0.80* and *r* = 0.40*). Among the determined phosphorus fractions, the content of available P was most closely correlated with the content of P bound in the carbonate fraction (*r* = 0.65*) and residual fraction (*r* = 0.60*) but less strongly correlated with the organic phosphorus fraction (*r* = 0.32*).

The content of P bound in each of the distinguished fractions also depended on the properties of the organic substances used for soil fertilisation and on the amounts of C-organic, N and P brought to the soil with these waste-based fertilisers (Table 3). The soil content of easily soluble phosphorus (F1) was negatively correlated with the content (*r* = − 0.39* and *r* = − 0.39*, respectively) and loads of N and P (*r* = − 0.41* and *r* = − 0.54*, respectively) added to the soil with the applied organic substances but was positively correlated with the C/N ratio C/N (*r* = 0.39*). The content of exchangeably bound phosphorus (F2) was positively correlated with the C/N (*r* = 0.37*), while the C/P and N/P ratios as well as the amounts of C-organic, N and P added to soil together with the organic substances had a negative effect on the soil content of this form of P (*r* = − 0.59*, *r* = − 80* and *r* = − 0.35*, respectively). Furthermore, the loads of N and P incorporated into soil with the organic phosphorus (F3) in soil. The content of P bound in the carbonate fraction was positively affected by the content of C-organic and its load added to the soil with the organic substances (*r* = 0.49* and *r* = 0.52*, respectively) applied as soil fertilisers, as well as by their content of nitrogen (*r* = 0.33*) and the C/P (*r* = 0.33*) and C/N (*r* = 0.35*) ratios. The quantities of C-organic and N introduced to the soil with the tested organic substances negatively influenced (*r* = − 0.46* and *r* = − 50*, respectively) the content of phosphorus accumulated in stable organic and mineral bonds (F5). Finally, the content of phosphorus bound in the residual fraction (F6) was positively correlated with the content of C-organic (*r* = 0.53*) and N (*r* = 0.64*) and with the content (*r* = 0.61*) and amount (0.41*) of P added to the soil with the organic substances while being negatively correlated with the C/P (*r* = − 0.33*) and N/P (*r* = − 0.38*) ratios.

**Table 3:** Coefficients of correlation *r* between selected properties of the organic substances and the content of determined phosphorus fractions

Selected properties of the organic substances	Phosphorus fractions					
	F1	F2	F3	F4	F5	F6
Content of selected macroelements						
C-org.	− 0.08	− 0.04	0.06	0.49*	− 0.02	0.53*
N-tot.	− 0.39*	− 0.30	0.24	0.33*	− 0.10	0.64*
P-tot.	− 0.39*	− 0.06	0.17	0.20	− 0.08	0.61*
C/P	0.26	− 0.46*	0.05	0.33*	− 0.23	− 0.33
C/N	0.39*	0.37*	− 0.17	0.35*	0.07	0.12
N/P	0.15	− 0.56*	0.10	0.23	− 0.27	− 0.38
Load of selected macroelements						
C-org.	− 0.10	− 0.59*	0.28	0.52*	− 0.46*	0.17
N-tot.	− 0.41*	− 0.80	0.42*	0.15	− 0.50*	0.10
P-tot.	− 0.54*	− 0.35*	0.35*	−0.06	− 0.27	0.41*

*Significant at *p* < 0.05, *n* = 36; *F1* easily soluble fraction; *F2* exchangeable fraction; *F3* organic fraction; *F4* carbonate fraction; *F5* stable organic-mineral and mineral bonds; *F6* residual fraction

## Conclusion

In comparison to the control, the NPK fertilisation as well as the soil fertilisation with organic substances raised the P-total content and the content of P bound in the fractions: F3 (organic fraction), F4 (carbonate fraction), F5 (stable organic and mineral bonds) and F6 (residual fraction). The NPK fertilisation decreased and the soil added CSSS increased the easily soluble P content (F1). The NPK fertilisation and the soil enrichment with organic substances (except CSSS and CMGW) led to a decrease in the amount of P bound in the exchangeable fraction (F2).

The percentages of P content in the distinguished fractions were as follows: F3 (34.7–39.7%)>F4 (21.8–27.5%)>F6 (19.7–25.3%)>F2 (6.5–12.8%)>F5 (4.2–8.4%)>F1 (1.6–2.7%). The analysed soil properties did not have any substantial influence on the content of easily soluble P (F1) or exchangeably bound P (F2). In turn, the organic P fraction (F3) was positively correlated with the soil’s CEC, and the widening of the C/P ratio had a negative effect on the content of this P fraction. The content of phosphorus determined in the carbonate fraction (F4) depended above all on the quantity of P-total and to a lesser extent on the CEC and C-organic content. The content of P in stable organic-mineral bonds (F5) correlated positively with the P-total content and soil pH while being negatively correlated with Hh and the C/P ratio. Furthermore, the content of P bound in the residual fraction (F6) depended primarily on the content of P-total in soil and, to a lesser degree, on the C-organic content and the C/P ratio. The content of P bound in the distinguished fractions also depended on the characteristics of the organic substances used to fertilise soil and on the amounts of C-organic, N and P added to soil with these substances.

The content of available P determined with the Egner-Riehm method positively correlated with the content of C-organic, P-total and soil CEC. Among the determined phosphorus fractions, the content of available P was most strongly correlated with the content of P bound in the carbonate fraction (F4) and residual fraction (F6) and, less strongly, with the organic phosphorus fraction (F3).

For environmental reasons and the possibility of leaching phosphorus from the soil, composted municipal sewage sludge and straw (CSSS) may pose the greatest threat due to the fact that it caused the largest accumulation of P labile forms in the soil (easily soluble P fraction and exchangeable P fraction). However, the highest amount of phosphorus was introduced into the soil from composted sewage sludge (CSS), and in this case the high surface runoff P can be a threat to the aquatic environment.
